# Supplementary material for: Early-life exercise induces immunometabolic epigenetic modification enhancing anti-inflammatory immunity in middle-aged male mice
Source: Nat Commun. 2024 Apr 10;15:3103. doi: 10.1038/s41467-024-47458-3 (PMC11006929; doi:10.1038/s41467-024-47458-3)

Supplementary Information

Early-life exercise induces immunometabolic epigenetic modification enhancing anti-inflammatory immunity in middle-aged male mice

Nini Zhang[1,2†], Xinpei Wang[1†], Mengya Feng[1,3], Min Li[1], Jing Wang[4], Hongyan Yang[1], Siyu He[1], Ziqi Xia[1], Lei Shang[5], Xun Jiang[2], Mao Sun[6], Yuanming Wu[6], Chaoxue Ren[7], Xing Zhang[1], Jia Li[1,5]*, Feng Gao[1]*

[1]Key Laboratory of Aerospace Medicine, Ministry of Education; School of Aerospace Medicine, Fourth Military Medical University, Xi’an, China. [2]Department of Pediatrics, Tangdu Hospital, Fourth Military Medical University, Xi’an, China. [3]Center for Mitochondrial Biology and Medicine, The Key Laboratory of Biomedical Information Engineering of Ministry of Education, School of Life Science and Technology, Xi’an Jiaotong University, Xi’an, China. [4]Department of Orthopedics, Xijing Hospital, Fourth Military Medical University, Xi'an, China. [5]Key Lab of Hazard Assessment and Control in Special Operational Environment, Ministry of Education; Department of Health Statistics, School of Public Health, Fourth Military Medical University, Xi'an, China. [6]Department of Biochemistry and Molecular Biology, Center for DNA Typing, Fourth Military Medical University, Xi'an China. [7]School of Sport and Health Science, Xi'an Physical Education University, Xi'an, China.

[†]These authors contributed equally: Nini Zhang, Xinpei Wang.

[*]**Corresponding authors:** Jia Li, jiali816@fmmu.edu.cn; Feng Gao, fgao@fmmu.edu.cn

Supplementary Figures 1-7

Supplementary Tables 1-6

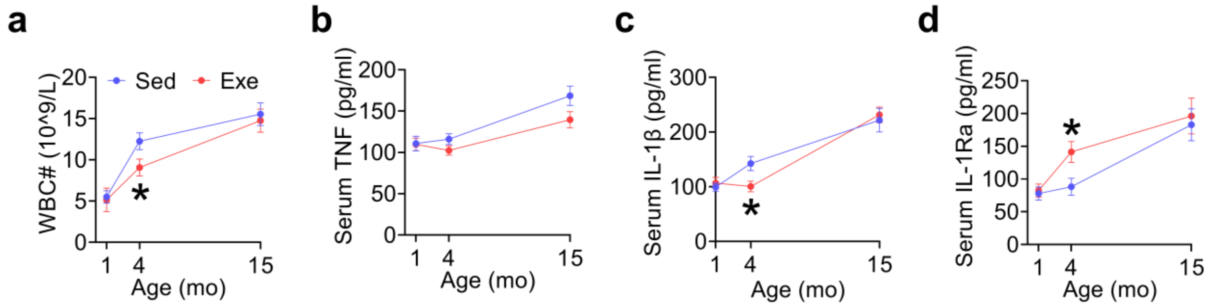

Supplementary Fig. 1 Effects of early-life exercise training on leucocytes and cytokines at different age. (a-d) White blood cell (WBC) count, serum TNF, IL-1β and IL-1Ra of Sed and Exe mice at different age (1, 4 and 15 months old). n=8 per group. Values are presented as mean ± SEM. Data are analyzed by using the two-way repeated measures ANOVA followed by Bonferroni's test. * $p < 0.05$; exact p values are listed in Source Data file. Sed=sedentary; Exe=exercise. Source data are provided as a Source Data file.

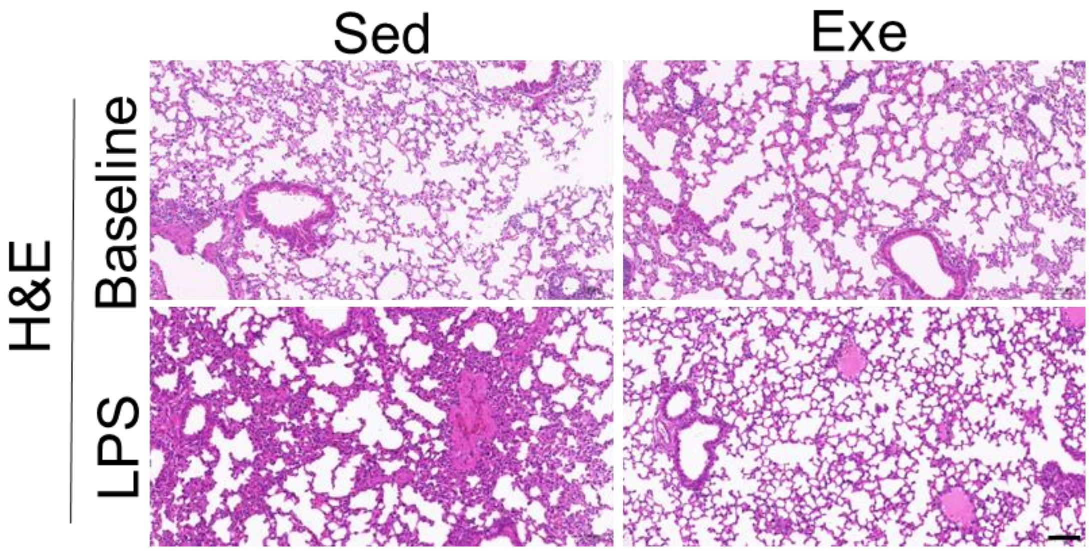

Supplementary Fig. 2 Early-life exercise reduced inflammatory injury in the lung of mice when exposed to LPS in middle age. Representative images of hematoxylin and eosin (H&E)-stained lung sections of mice at baseline and 48 hours after LPS infection. Scale bar, 50 μm.

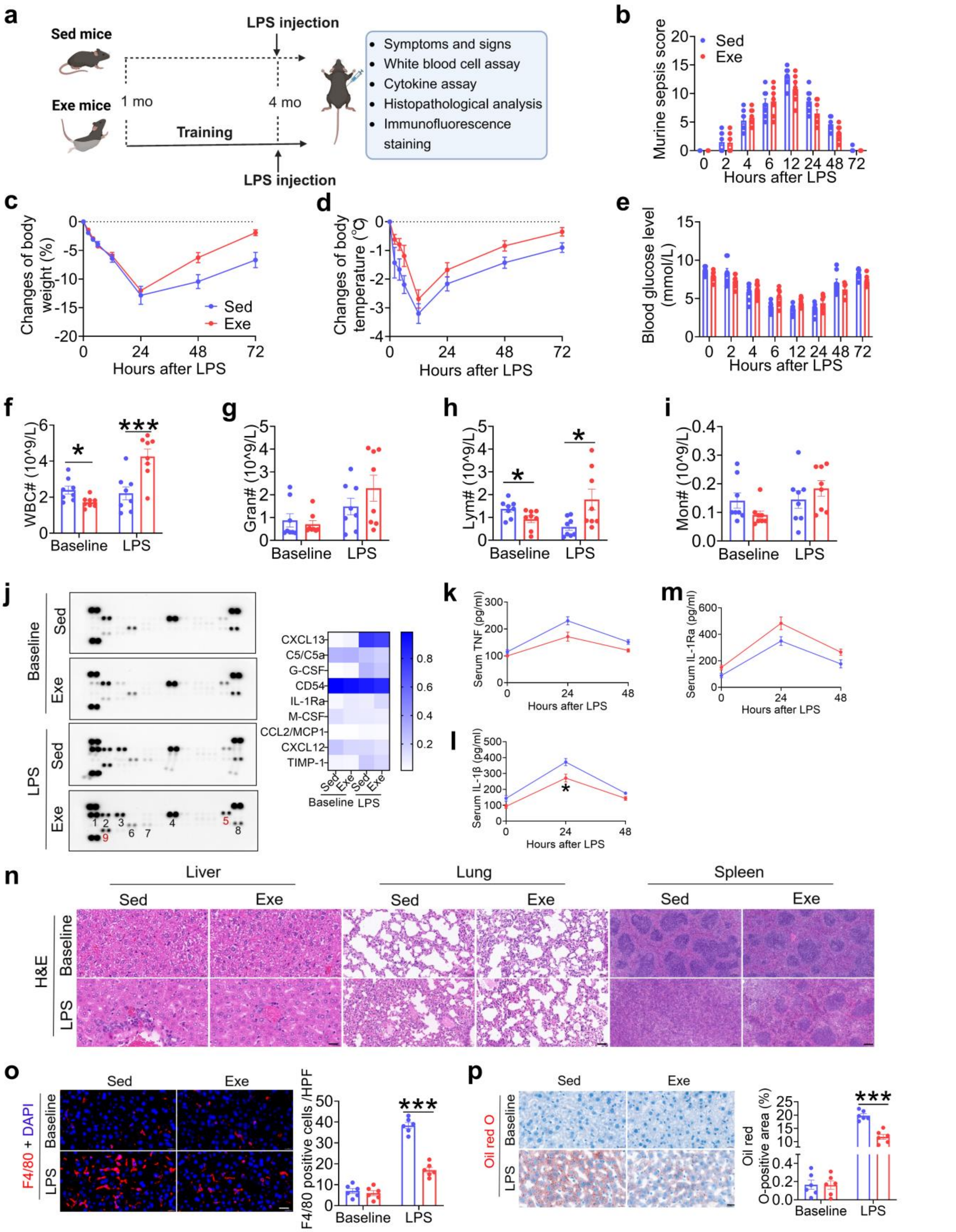

Supplementary Fig. 3 Three-month swim training protected vital organs against LPS-induced sepsis in mice. a Experimental outline (created with Biorender.com). C57BL/6 mice were subjected

to swimming exercise for 3 months starting from 1-month-old. After 24 hours post 3-month exercise training, mice were subjected to a single i.p. injection of LPS. The clinical severity score **b**, changes of body weight **c**, changes of body temperature **d** and blood glucose level **e** in different time points (0, 2, 4, 6, 12, 24, 48 and 72 hours) after injection. **f-i** The quantity of WBCs, granulocytes, lymphocytes and monocytes in the blood at baseline and 6 hours after LPS infection. **j** Cytokine array analysis in the serum at baseline and 24 hours after LPS infection. **k-m** Serum TNF, IL-1β and IL-1Ra levels at 0, 24 and 48 hours after LPS infection. n=6 per group. **(N)** Representative images of hematoxylin and eosin-stained (H&E) liver (scale bar, 20 μm), lung (scale bar, 50 μm) and spleen (scale bar, 50 μm) sections of mice at baseline and 48 hours after LPS infection. **o** Representative images and quantitate analysis of F4/80 (red) staining in the liver sections of mice at baseline and 48 hours after LPS infection. n=6 per group. Scale bar, 20 μm. **p** Representative images and quantitate analysis of Oil red O-staining in the liver sections of mice at baseline and 48 hours after LPS infection. n=6 per group. Scale bar, 20 μm. Values are presented as mean ± SEM. Data are analyzed using the two-way repeated measures ANOVA followed by Bonferroni's test (**b-e, k-l**) or unpaired, two-tailed Student's t-test (f, h, o-p) or Mann-Whitney U test (g, i). * $p < 0.05$; ** $p < 0.01$; *** $p < 0.001$; exact p values are listed in Source Data file. Sed=sedentary; Exe=exercise. Source data are provided as a Source Data file.

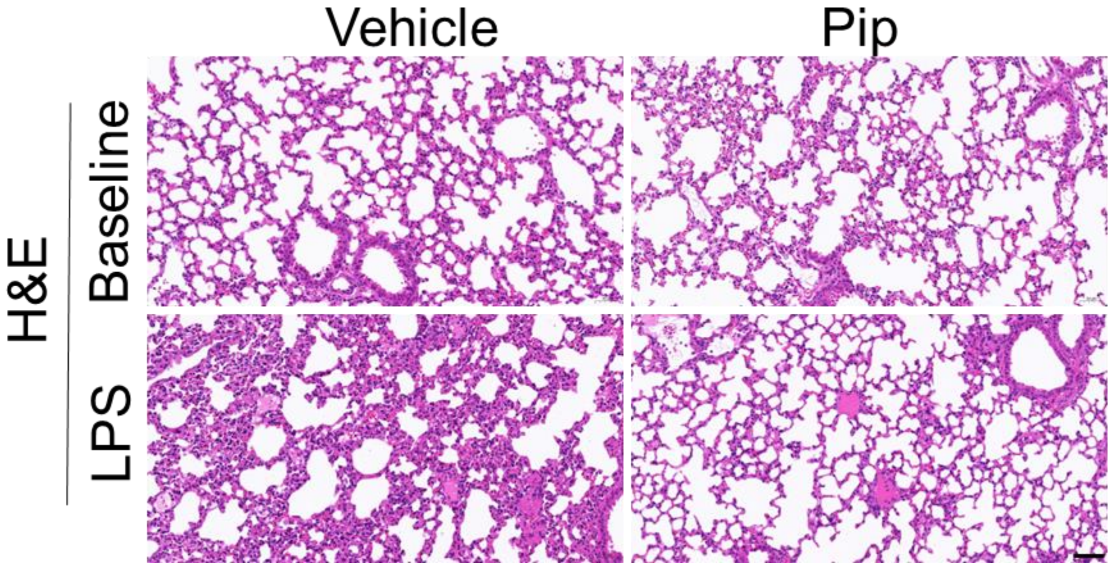

Supplementary Fig. 4 Pipecolic acid administration reduced inflammatory injury in the lung of mice exposed to LPS. Representative images of hematoxylin and eosin (H&E)-stained lung sections of mice at baseline and 48 hours after LPS infection. Scale bar, 50 μm. Pip=pipecolic acid.

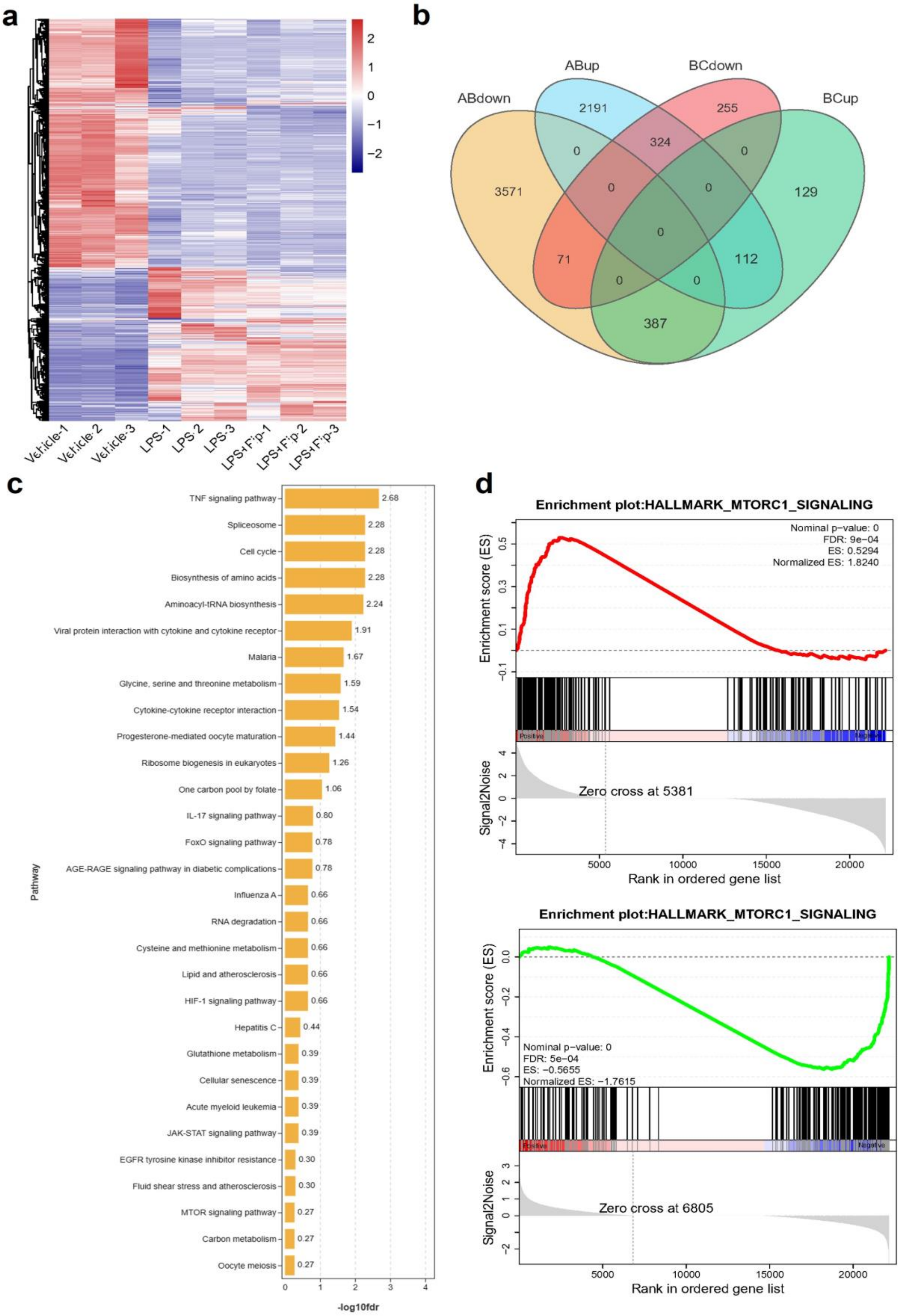

Supplementary Fig. 5 Pipecolic acid inhibited LPS-induced mTORC1 signaling in macrophages. a Gene expression heatmap of DEGs (n=3). **b** Number of genes significantly different

in LPS vs. vehicle control and/or in LPS+Pip vs. LPS in BMDMs (n=3). A=Vehicle; B=LPS; C=LPS+Pip. **c** KEGG analysis of 324 DEGs that were upregulated in BMDMs with LPS stimulation but downregulated after pipecolic acid treatment. **d** GSEA analysis of mTORC1 signaling pathway. Pip=pipecolic acid.

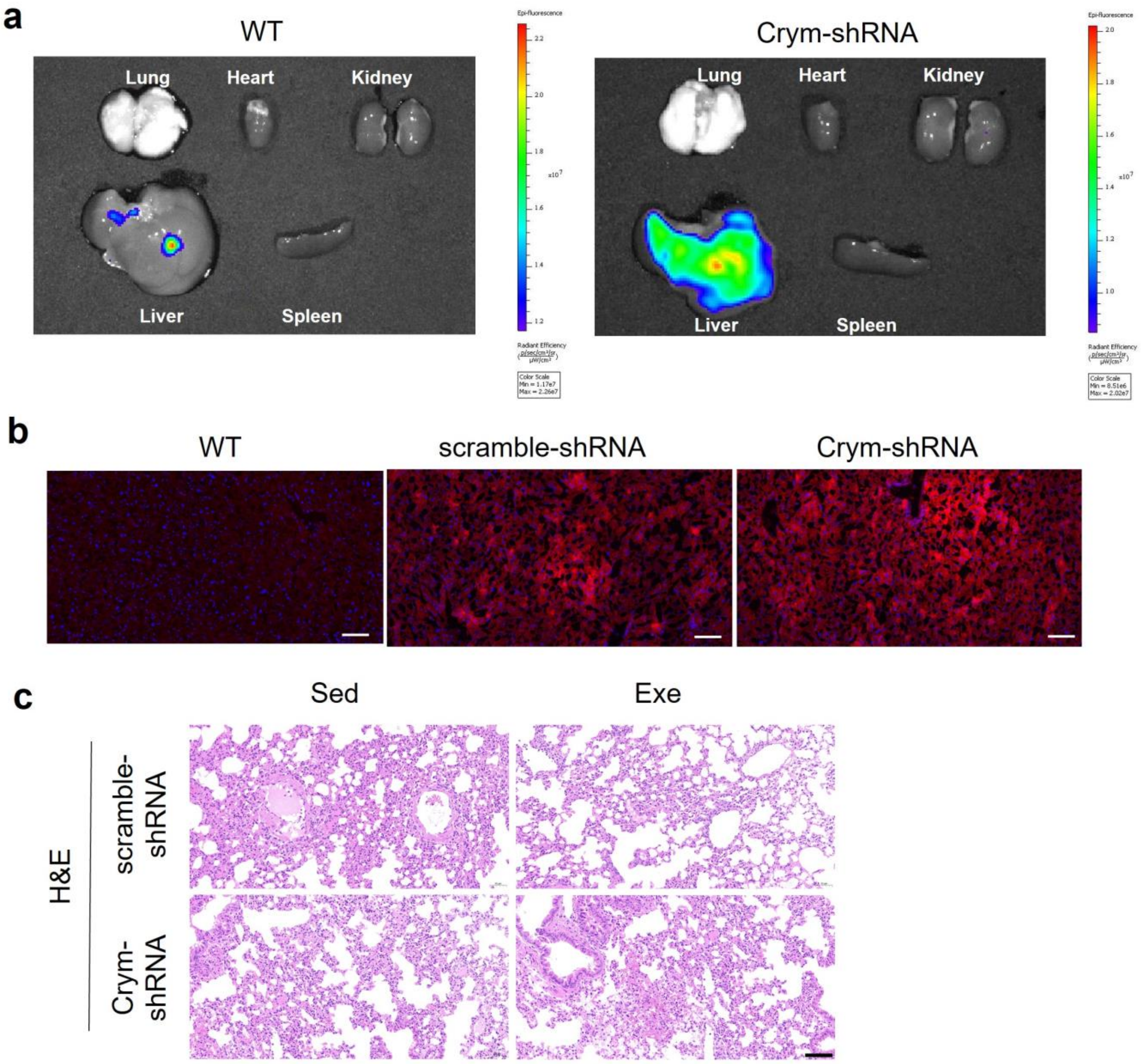

Supplementary Fig. 6 Determination of transfection efficiency in the liver. a Representative images of mCherry fluorescence in different tissues in wild-type mice and mice injected with the serotype 8 adeno-associated virus (AAV8) vector carrying shRNA targeting Crym (Crym-shRNA). **b** mCherry, an indicator for the AAV8 vector, was detectable by confocal microscopy in liver transfected with AAV8. **c** Representative images of hematoxylin and eosin (H&E)-stained lung sections of mice at 48 hours after LPS infection. Scale bar, 50 μm.

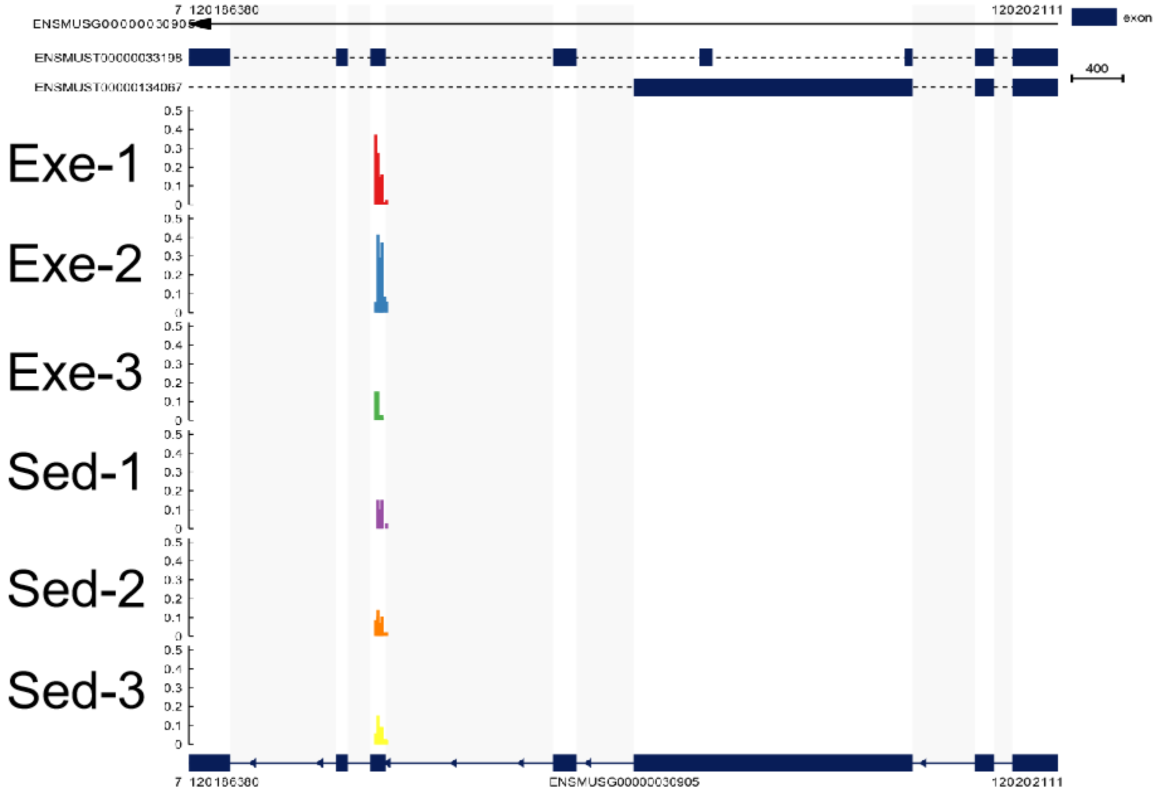

Supplementary Fig. 7 DNA methylation level of *Crym* gene in the liver of Exe and Sed mice. The representative images of DNA methylation of *Crym* gene in the liver of Sed and Exe mice aged at 15 months (n=3 for each group). The image was obtained from Whole Genome Bisulfite Sequencing (WGBS). The number on the Y axis indicates RPM (Reads per million), a parameter that measure the degree of enrichment. Sed=sedentary; Exe=exercise.

Supplementary Table 1 Murine Sepsis Score (MSS).

Variable	Score and description
Appearance	0. Coat is smooth
	1. Patches of hair piloerected
	2. Majority of back is piloerected
	3. Piloerection may or may not be present, mouse appears “puffy”
	4. Piloerection may or may not be present, mouse appears emaciated
Level of consciousness	0. Mouse is active
	1. Mouse is active but avoids standing upright
	2. Mouse activity is noticeably slowed. The mouse is still ambulant
	3. Activity is impaired. Mouse only moves when provoked, movements have a tremor
	4. Activity severely impaired. Mouse remains stationary when provoked, with possible tremor
Activity	0. Normal amount of activity. Mouse is involved in any of the following activities: Eating, drinking, climbing, running, fighting
	1. Slightly suppressed activity. Mouse is moving around bottom of cage
	2. Suppressed activity. Mouse is stationary with occasional investigative movements
	3. No activity. Mouse is stationary
	4. No activity. Mouse experiencing tremors, particularly in the hind legs
Response to stimulus	0. Mouse responds immediately to auditory stimulus or touch
	1. Slow or no response to auditory stimulus, strong response to touch (moves to escape)
	2. No response to auditory stimulus, moderate response to touch (moves a few steps)
	3. No response to auditory stimulus, mild response to touch (no locomotion)
	4. No response to auditory stimulus. Little or no response to touch. Cannot right itself if pushed over

Eyes	0. Open
	1.Eyes not fully open, possibly with secretions
	2. Eyes at least half closed, possibly with secretions
	3. Eyes half closed or more, possibly with secretions
	4. Eyes closed or milky
Respiration rate	0. Normal, rapid mouse respiration
	1. Slightly decreased respiration (rate not quantifiable by eye)
	2. Moderately reduced respiration (rate at the upper range of quantifying by eye)
	3. Severely reduced respiration (rate easily countable by eye, 0.5 s between breaths)
	4. Extremely reduced respiration (>1 s between breaths)
Respiration quality	0. Normal
	1. Brief periods of labored breathing
	2. Labored breathing, no gasping
	3. Labored with intermittent gasping
	4. Gasping

146 **Supplementary Table 2 Differentially concentrated metabolites of serum in 4-month-old mice.**

Name	Log2_FC(case mean/control mean)	P value	FDR	VIP
LysoPC(22:6(4Z,7Z,10Z,13Z,16Z,19Z))	-0.469246443	2.28E-06	0.000650	17.24
LysoPC(20:4(5Z,8Z,11Z,14Z))	-0.309256502	0.007092532	0.115503	10.61
4-(trimethylazaniumyl)butanoate	-0.428402948	5.23E-05	0.005813	6.66
8-Hydroxypinoresinol 4-glucoside	-0.89700277	0.003308631	0.073488	5.98
L-Glutamic acid	-0.584110383	0.000408211	0.021799	4.33
**Pipecolic acid**	**0.480569672**	**0.035910594**	**0.291683**	**3.19**
1-Methylhistidine	-0.330394428	0.00302549	0.069318	2.93
6-amino-1H-pyrimidin-2-one	-0.210864781	0.02241995	0.227601	2.85
PC(22:6(4Z,7Z,10Z,13Z,16Z,19Z)/18:3(6Z,9Z,12Z))	-0.097735599	0.048334545	0.334517	2.02
13-chloro-2-piperidin-4-ylidene-4-azatricyclo[9.4.0.03,8]pentadeca-1(11),3(8),4,6,12,14-hexaene	-0.359038516	0.026379347	0.247615	1.73
2-(1H-imidazol-5-yl)acetic acid	-0.331847781	0.005249074	0.095826	1.62
Netilmicin	-1.787031359	0.000282823	0.017316	1.60
1-(1-Pyrrolidinyl)-2-propanone	0.644425926	0.034765212	0.287172	1.53
Glycerylphosphorylethanolamine	-0.690026499	0.003045045	0.069566	1.50
1-Pyrroline-4-hydroxy-2-carboxylate	-0.281631881	0.01056608	0.148372	1.32
Montecristin	-0.769968343	0.001007683	0.036625	1.25
5-Aminoimidazole ribonucleotide	-0.352058443	0.00094352	0.035090	1.25
(2S)-5-oxopyrrolidine-2-carboxylic acid	-0.2931504	0.037033398	0.296119	1.17
PA(18:1(9Z)/20:5(5Z,8Z,11Z,14Z,17Z))	0.892742013	6.05E-05	0.006362	1.12
6-Deoxohomodolichosterone	0.61709148	0.003367733	0.074183	1.04

147 *p* values are determined by unpaired, two-tailed Student's t-test, FDR were subsequently determined
148 by Benjamini-Hochberg correction method. Abbreviations: VIP, variable importance in projection;
149 FC, fold change; FDR, false discovery rate.

150

Supplementary Table 3 Differentially concentrated metabolites of serum in 15-month-old mice.

Name	Log2_FC(case mean/control mean)	P value	FDR	VIP
L-Arginine	-0.26842	0.022466	0.432115	11.22
N1-Methyl-4-pyridone-3-carboxamide	0.749909	0.022357	0.432115	7.61
**Pipecolic acid**	**0.815227**	**0.011061**	**0.375526**	**5.84**
Butyrylcarnitine	-0.58956	0.048083	0.491372	3.75
5-Ethyl-2,4-dimethyloxazole	1.718371	0.031207	0.432147	3.68
L-Tyrosine	-0.94037	0.004117	0.356110	3.66
L-2-Amino-3-methylenehexanoic acid	1.062436	0.003145	0.350528	2.95
3-Methylglutarylcarnitine	0.905903	0.019871	0.426313	2.90
(2R,3R,4R)-2-Amino-4-hydroxy-3-methylpentanoic acid	-0.84833	0.003346	0.350528	2.70
6-Chloro-N-(1-methylethyl)-1,3,5-triazine-2,4-diamine	-0.51918	0.047626	0.490491	2.69
apo-[3-methylcrotonoyl-CoA:carbon-dioxide ligase (ADP-forming)]	0.802807	0.047819	0.491372	2.66
SM(d18:1/22:0)	0.491436	0.005202	0.366865	2.65
L-Lysine	-0.30008	0.021755	0.432115	2.65
PC(22:5(7Z,10Z,13Z,16Z,19Z)/14:0)	-0.21096	0.024642	0.432115	2.38
Mesalazine	0.692351	0.027319	0.432115	2.12
L-cis-3-Amino-2-pyrrolidinecarboxylic acid	-0.92148	0.027681	0.432115	2.05
2,2,6,6-Tetramethyl-4-piperidinone	0.714119	0.001805	0.350528	1.94
2-Pyridylacetic acid	0.382474	0.010914	0.375526	1.92
PC(24:1(15Z)/14:1(9Z))	-0.37456	0.040562	0.470813	1.88
2-Phenylacetamide	-1.05102	0.003955	0.356110	1.82

p values are determined by unpaired, two-tailed Student's t-test, FDR were subsequently determined by Benjamini-Hochberg correction method. Abbreviations: VIP, variable importance in projection;

FC, fold change; FDR, false discovery rate.

Supplementary Table 4 Differentially concentrated metabolites of liver in 15-month-old mice.

Name	Log2_FC(case mean/control mean)	P value	FDR	VIP
PC(22:6(4Z,7Z,10Z,13Z,16Z,19Z)/16:0)	0.420746	0.012218	0.300430	11.99
Adenine	0.650732	0.033853	0.406885	8.97
5-Aminoimidazole ribonucleotide	-1.49771	0.049779	0.444688	6.63
Methoxypyrazine	-0.67912	0.026163	0.373824	6.41
Glutathione	-2.83202	0.02865	0.384977	4.79
**Pipecolic acid**	**0.820006**	**0.021898**	**0.357343**	**4.60**
PE(16:0/22:6(4Z,7Z,10Z,13Z,16Z,19Z))	-0.70498	0.04741	0.442861	4.10
Pyrrolidonecarboxylic acid	-1.21481	0.000488	0.078683	3.88
PC(16:0/16:0)	-0.22273	0.030587	0.391350	2.97
N-a-Acetyl-L-arginine	1.24795	0.002213	0.149389	2.54
2-Methyl-5-propyloxazole	2.39478	0.008706	0.279332	2.48
PC(22:5(4Z,7Z,10Z,13Z,16Z)/20:5(5Z,8Z,11Z,14Z,17Z))	-0.75606	0.032769	0.401953	2.06
N-Alpha-acetyllysine	-0.55985	0.028988	0.384977	2.06
Cholesterol	1.013156	0.00012	0.037965	1.79
N-methyl-L-glutamic Acid	-0.90748	0.025545	0.372285	1.79
(+)-2,3-Dihydro-3-methyl-1H-pyrrole	0.669816	0.027761	0.382078	1.58
PC(20:2(11Z,14Z)/15:0)	-0.40051	0.010918	0.298467	1.57
Sedoheptulose	-2.19287	0.049446	0.443725	1.45
PE(22:6(4Z,7Z,10Z,13Z,16Z,19Z)/18:0)	0.254515	0.032495	0.401953	1.41
Isotheaflavin	-0.21678	0.037721	0.424625	1.40

p values are determined by unpaired, two-tailed Student's t-test, FDR were subsequently determined by Benjamini-Hochberg correction method. Abbreviations: VIP, variable importance in projection; FC, fold change; FDR, false discovery rate.

Supplementary Table 5 Characteristics of participants.

Characteristics	Untrained (n=21)	Trained (n=18)	P value
Age (years)	16.57±3.63	15.78±1.31	0.798
Weight (kg)	67.19±7.93	61.94±6.79	0.034
Height (m)	1.76±0.07	1.74±0.13	0.923
BMI (kg/m2)	21.68±1.56	19.95±1.45	0.001

p values are determined by unpaired, two-tailed Student's t-test.

Supplementary Table 6 Sequences of gene-specific primers.

Gene		Sequences (5’-3’)
Actb	Forward	CTGTCCACCTTCCAGCAGATGT
	Reverse	CGCAACTAAGTCATAGTCCGCC
Crym	Forward	ATGAGGCAAGCGGTGCTGTATG
	Reverse	GTGGTCTTCTCACAGTGTGCAG
Aass	Forward	GACCAGCAAATTATTCACGACA
	Reverse	TTGTGATGCATCGGATAACAAC
Pycr1	Forward	GTGATGTGCTCTTCCTGGCTGTG
	Reverse	ATGTGCCTGTCCTCAATGTTCGC
Pipox	Forward	GGCTTATCCAGAGGACTTCTAC
	Reverse	AAAGATACTCATGGTCGATCCC
Hykk	Forward	AGGTCATTCGGATGTTCAAGGAAGAAG
	Reverse	AGAGGCTGACTTGCTGAGGTCTAC
Phykpl	Forward	CATGACAACATCGTGGACTATG
	Reverse	GTACTGTCGAGCTAGTCTCAAG
Slc25a29	Forward	GTCCATCATCAAGCAGGAGAGTGTG
	Reverse	GGAACTGATTGAGTGGTGAGTCTTGG
36B4	Forward	GAGACTGAGTACCTTCCCAC
	Reverse	ATGCAGATCAGCCAGG
TNF	Forward	GAGTGACAAGCCTGTAGCC
	Reverse	CTCCTGGTATGAGATAGCAAA
IL-1β	Forward	CCTCGTGCTGTCGGACCCATA
	Reverse	CAGGCTTGTGCTCTGCTTGTGA
iNOS	Forward	CACCAAGCTGAACTTGAGCG
	Reverse	CGTGGCTTTGGGCTCCTC
Kmt2a	Forward	CGATGACAACCGACAGTGTGCA
	Reverse	GCTGACCACAAAGCACAGTTCAC

Kmt2b	Forward	GGAAGCCAGATGAAAGGACTCC
	Reverse	TGGTCCAAGGATGGAGGCAACA
Kmt2c	Forward	CCTATCCTCCAGAGGTTGCTGG
	Reverse	TTTGCTGAGGCACATGGAAGCG
Kmt2d	Forward	CACTATAAACGGCCCCATACC
	Reverse	TGACTTGGAGTGCACAAACTG
Kmt2e	Forward	GACCATCACAGGGTTTGTGG
	Reverse	CTATGCTCATGACGTTCGCC
Setd1a	Forward	CCAGGAGTCTTCCTCAGAGAAG
	Reverse	GACGAGTCACTGTCCTCATCCT
Setd1b	Forward	GACACCAAAGGGGAAACTCG
	Reverse	GTCTCGCTCACAATCGGAGA

Supplementary Fig. 3j

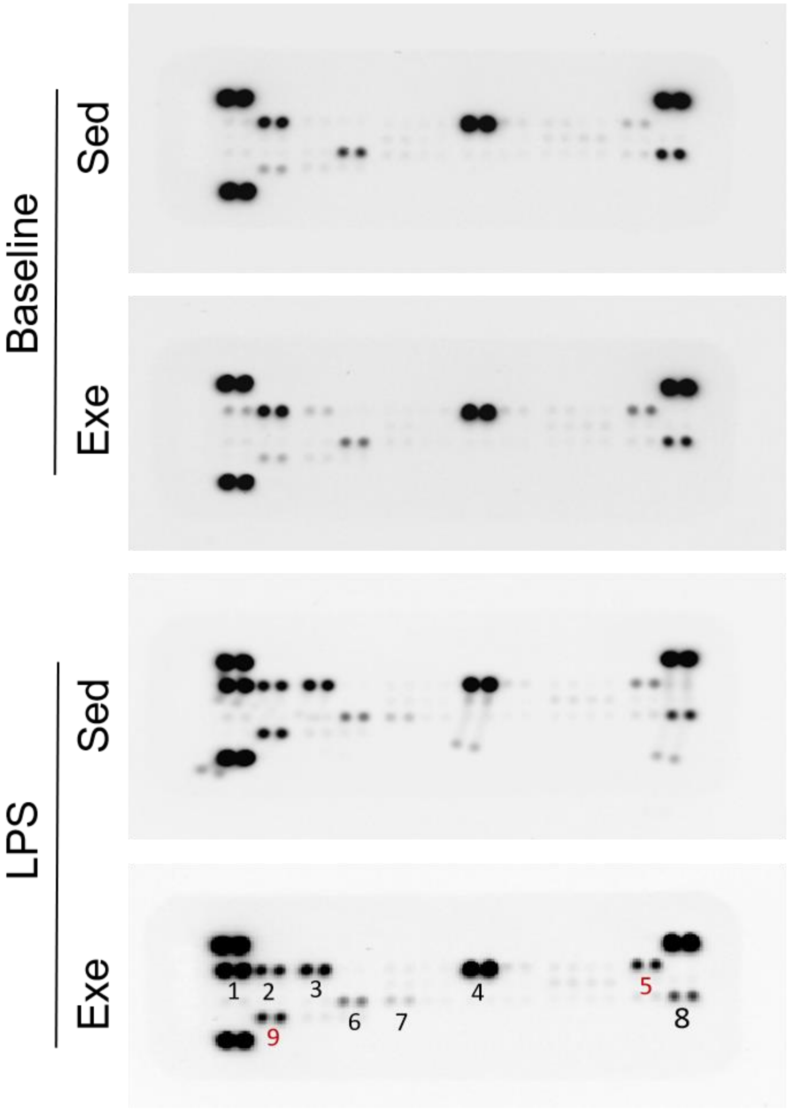

Supplement: Supplementary file 1 — Supplementary information [file 41467_2024_47458_MOESM1_ESM.pdf]
