# Supplementary material for: Early-life exercise induces immunometabolic epigenetic modification enhancing anti-inflammatory immunity in middle-aged male mice
Source: Nat Commun. 2024 Apr 10;15:3103. doi: 10.1038/s41467-024-47458-3 (PMC11006929; doi:10.1038/s41467-024-47458-3)
Supplement: Supplementary file 3 — Reporting Summary [file 41467_2024_47458_MOESM3_ESM.pdf]

nature portfolio

Corresponding author(s): Feng Gao

Last updated by author(s): May 16, 2024

Reporting Summary

Nature Portfolio wishes to improve the reproducibility of the work that we publish. This form provides structure for consistency and transparency in reporting. For further information on Nature Portfolio policies, see our Editorial Policies and the Editorial Policy Checklist.

Statistics

For all statistical analyses, confirm that the following items are present in the figure legend, table legend, main text, or Methods section.

n/a	Confirmed	
☐	☒	The exact sample size (*n*) for each experimental group/condition, given as a discrete number and unit of measurement
☐	☒	A statement on whether measurements were taken from distinct samples or whether the same sample was measured repeatedly
☐	☒	The statistical test(s) used AND whether they are one- or two-sided<br>*Only common tests should be described solely by name; describe more complex techniques in the Methods section.*
☒	☐	A description of all covariates tested
☐	☒	A description of any assumptions or corrections, such as tests of normality and adjustment for multiple comparisons
☐	☒	A full description of the statistical parameters including central tendency (e.g. means) or other basic estimates (e.g. regression coefficient) AND variation (e.g. standard deviation) or associated estimates of uncertainty (e.g. confidence intervals)
☐	☒	For null hypothesis testing, the test statistic (e.g. *F*, *t*, *r*) with confidence intervals, effect sizes, degrees of freedom and *P* value noted<br>*Give P values as exact values whenever suitable.*
☒	☐	For Bayesian analysis, information on the choice of priors and Markov chain Monte Carlo settings
☒	☐	For hierarchical and complex designs, identification of the appropriate level for tests and full reporting of outcomes
☒	☐	Estimates of effect sizes (e.g. Cohen's *d*, Pearson's *r*), indicating how they were calculated

Our web collection on statistics for biologists contains articles on many of the points above.

Software and code

Policy information about availability of computer code

Data collection	Data were acquired by Element Ht5 Auto Hematology analyzer (Heska, Canada); Nikon E200 light microscope (Nikon, Tokyo, Japan); Inverted confocal microscope (Zeiss LSM 800); Denovix DS-11 spectrophotometer (Denovix, Wilmington, USA); ECL system (Image Quant350, GE Healthcare, UK); UHPLC system (Agilent Technologies, Santa Clara, CA, USA, #1290); tandem mass spectrometry (UPLC-MS/MS) system (ACQUITY UPLC-Xevo TQ-S, Waters Corp., MA, USA); Agilent 2100 Bioanalyzer (Agilent Technologies, Palo Alto, CA, USA).
Data analysis	Data were analyzed by Image-Pro Plus software (version 6.0, Media cybernetics) ; Mass-Lynx software (ver. 4.1, Waters Corp.); SPSS software (version 26.0); STAR software (version 2.5.3a); RSeQC (version 2.6); MACS2 software (Version 2.1.1); Prism 9.5.1 software (Graphpad, USA)

For manuscripts utilizing custom algorithms or software that are central to the research but not yet described in published literature, software must be made available to editors and reviewers. We strongly encourage code deposition in a community repository (e.g. GitHub). See the Nature Portfolio guidelines for submitting code & software for further information.

Data

Policy information about availability of data

All manuscripts must include a data availability statement. This statement should provide the following information, where applicable:

- Accession codes, unique identifiers, or web links for publicly available datasets
- A description of any restrictions on data availability
- For clinical datasets or third party data, please ensure that the statement adheres to our policy

All data are available within the Article and Supplementary Files, or available from the corresponding authors on reasonable request. RNA sequence data that support the findings of this study have been deposited in GSA with the accession code “CRA011636”. Chip-Seq data generated in this study have been deposited in the Gene Expression Omnibus database under accession code “GSE255161”. The metabolome data generated in this study have been deposited in the OMIX, under accession code “OMIX006004”. Source data are provided with this paper.

Human research participants

Policy information about studies involving human research participants and Sex and Gender in Research.

Reporting on sex and gender	Sex is reported on self-identification. All the participants are male.
Population characteristics	A total of 39 healthy young (aged 16.21±2.80 years) were included: 18 young highly-trained athletes (trained group) and 21 inactive healthy individuals (untrained group), all of whom are male, all non-smokers with no history of cardiac arrhythmia and not taking any cardiovascular medication. Ethnicity of participants was determined by self-report. Participants in this study are all Chinese.
Recruitment	Participants were recruited through campus advertisements from physical education students at Xi’an Physical Education University who volunteered to have blood samples collected for the purpose of collecting relevant data for this study.
Ethics oversight	The study was conducted according to Declaration of Helsinki principles and were approved by the Human Research Ethics Committee of Xi’an Physical Education University (Approval No. XAIPE2023011). All participants signed a consent form after reading information about the study and having the procedures explained to them.

Note that full information on the approval of the study protocol must also be provided in the manuscript.

Field-specific reporting

Please select the one below that is the best fit for your research. If you are not sure, read the appropriate sections before making your selection.

☒ Life sciences ☐ Behavioural & social sciences ☐ Ecological, evolutionary & environmental sciences

For a reference copy of the document with all sections, see nature.com/documents/nr-reporting-summary-flat.pdf

Life sciences study design

All studies must disclose on these points even when the disclosure is negative.

Sample size	No statistical methods were used to predetermine sample sizes. We used generally accepted sample sizes in accordance to our own previous experiences with reproducible differences between conditions indicating that the chosen sample sizes were sufficient. Our sample sizes are similar to those generally employed in the life sciences field.<br>All experiments were performed with at least 3 independent biological replicates per condition. At least 6 animals were included in each group for in vivo analyses. The exact number of samples are indicated on scatter plots in each figure and were elucidated in the corresponding legends.
Data exclusions	We did not exclude any data.
Replication	Data were obtained in at least three biologically independent replicates and the number of animals or experiments were described in corresponding figure legends.
Randomization	All C57BL/6J mice were divided into the control or experimental groups using a completely randomized design with random digits table. Cells were randomized into different groups before treatment. Human participants were recruited by the recruitment standards and no randomization was applied.
Blinding	Investigators were not blinded in conducting animal experiments or collecting samples for analysis of body weight, glucose tolerance and temperature changes. For cellular experiments, data collection was not blinded. Researchers were blinded during data analysis.

Reporting for specific materials, systems and methods

We require information from authors about some types of materials, experimental systems and methods used in many studies. Here, indicate whether each material, system or method listed is relevant to your study. If you are not sure if a list item applies to your research, read the appropriate section before selecting a response.

Materials & experimental systems

n/a	Involved in the study
☐	☒ Antibodies
☒	☐ Eukaryotic cell lines
☒	☐ Palaeontology and archaeology
☐	☒ Animals and other organisms
☒	☐ Clinical data
☒	☐ Dual use research of concern

Methods

n/a	Involved in the study
☐	☒ ChIP-seq
☒	☐ Flow cytometry
☒	☐ MRI-based neuroimaging

Antibodies

Antibodies used

Western blot: phospho-mTOR (Ser2448) (1:1000, 5536), mTOR (1:1000, 2983), phospho-p70S6K (Thr389) (1:1000, 9234), total p70S6K (1:1000, 2708), phospho-S6 ribosomal protein (Ser240/244) (1:1000, 2215), total S6 ribosomal protein (1:1000, 2217), H3K4me3 (1:1000, 9751), H3K27me3 (1:1000, 9733), H3K27ac (1:1000, 8173), histone-H3 (1:2000, 4499), α-Tubulin (1:1000, 2125) and β-actin (1:1000, 4970) were purchased from Cell Signaling Technology (Danvers, MA, USA). Aass (1:2000, sc-365417) and Crym (1:500, sc-376687) were purchased from Santa Cruz Biotechnology (Dallas, Texas, USA). Pycr1 (1:500, ab279385) was purchased from Abcam (Cambridge, UK). SETD1A (1:1000, 67936-1-lg) were purchased from Proteintech (Wuhan, China). HRP-conjugated goat-anti-rabbit secondary antibody (1:5000, ab6721) and HRP-conjugated goat-anti-mouse secondary antibody (1:5000, ab6789) were purchased from Abcam (Cambridge,UK).
Chip-seq and Chip: H3K4me3 (1:50, #9751, Cell Signaling Technology) antibodies; H3 (1:50, #4620, Cell Signaling Technology); rabbit IgG (1:100, #2729, Cell Signaling Technology)

Validation

All antibodies were purchased from commercial vendors and were validated by manufactures, other studies and/or in this study. We also provided associated links as below.
phospho-mTOR (Ser2448), https://www.cellsignal.cn/products/primary-antibodies/phospho-mtor-ser2448-d9c2-xp-174-rabbit-mab/5536
mTOR, https://www.cellsignal.cn/products/primary-antibodies/mtor-7c10-rabbit-mab/2983
phospho-p70S6K (Thr389), https://www.cellsignal.cn/products/primary-antibodies/phospho-p70-s6-kinase-thr389-108d2-rabbit-mab/9234
total p70S6K, https://www.cellsignal.cn/products/primary-antibodies/p70-s6-kinase-49d7-rabbit-mab/2708
phospho-S6 ribosomal protein, https://www.cellsignal.cn/products/primary-antibodies/phospho-s6-ribosomal-protein-ser240-244-antibody/2215
total S6 ribosomal protein, https://www.cellsignal.cn/products/primary-antibodies/s6-ribosomal-protein-5g10-rabbit-mab/2217
H3K4me3, https://www.cellsignal.cn/products/primary-antibodies/tri-methyl-histone-h3-lys4-c42d8-rabbit-mab/9751
H3K27me3, https://www.cellsignal.cn/products/primary-antibodies/tri-methyl-histone-h3-lys27-c36b11-rabbit-mab/9733
H3K27ac, https://www.cellsignal.cn/products/primary-antibodies/acetyl-histone-h3-lys27-d5e4-xp-174-rabbit-mab/8173
histone-H3, https://www.cellsignal.cn/products/primary-antibodies/histone-h3-d1h2-xp-rabbit-mab/4499
α-Tubulin, https://www.cellsignal.cn/products/primary-antibodies/a-tubulin-11h10-rabbit-mab/2125
β-actin, https://www.cellsignal.cn/products/primary-antibodies/b-actin-13e5-rabbit-mab/4970
Aass, https://www.scbt.com/zh/p/aass-antibody-b-7?requestFrom=search
Crym, https://www.scbt.com/p/mu-crystallin-antibody-f-11?requestFrom=search
Pycr1, https://www.abcam.cn/products/primary-antibodies/pycr1-antibody-oti4f2-ab279385.html
SETD1A, https://www.ptgcn.com/products/SETD1A-Antibody-67936-1-Ig.htm
HRP-conjugated goat-anti-rabbit secondary antibody, https://www.abcam.cn/products/secondary-antibodies/goat-rabbit-igg-hl-hrp-ab6721.html
HRP-conjugated goat-anti-mouse secondary antibody, https://www.abcam.cn/products/secondary-antibodies/goat-mouse-igg-hl-hrp-ab6789.html

Animals and other research organisms

Policy information about studies involving animals; ARRIVE guidelines recommended for reporting animal research, and Sex and Gender in Research

Laboratory animals

One-month-old male C57BL/6J mice were obtained from the Animal Center of Fourth Military Medical University.

Wild animals

No wild animals were used in this study.

Reporting on sex

Only male mice were used in this study. The sole use of male mice has been indicated in the title, abstract and methods of the manuscript.

Field-collected samples | No field-collected samples were used in this study.

Ethics oversight | Laboratory Animal Welfare and Ethics Committee of Fourth Military Medical University (Approval No. 20180303)

Note that full information on the approval of the study protocol must also be provided in the manuscript.

ChIP-seq

Data deposition

☒ Confirm that both raw and final processed data have been deposited in a public database such as GEO.

☒ Confirm that you have deposited or provided access to graph files (e.g. BED files) for the called peaks.

Data access links
May remain private before publication.

https://www.ncbi.nlm.nih.gov/geo/submission/update/?acc=GSE255161

Files in database submission

For raw files:
Ex_1_IP.R1.fq.gz
Ex_1_Input.R1.fq.gz
Sed_3_IP.R1.fq.gz
Sed_3_Input.R1.fq.gz

For processed files:
Ex_1_IP.bw
Ex_1_Input.bw
Sed_1_IP.bw
Sed_1_Input.bw

Genome browser session
(e.g. UCSC)

N/A

Methodology

Replicates

One replicate for exercised group and one replicate for sedentary group.

Sequencing depth

Ex_1_IP: total reads, 77888004; unique reads, 72811002;
Ex_1_Input: total reads, 81157828; unique reads, 70285307;
Sed_3_IP: total reads, 68903070; unique reads, 64290562;
Sed_3_Input: total reads, 73343254; unique reads, 65613068.

Antibodies

H3K4me3 (1:50, #9751, Cell Signaling Technology) antibodies

Peak calling parameters

The MACS2 software (Version 2.1.1) was used for peak calling. The bedtools (Version 2.25.0) was used for peaks annotation and peak distribution analysis. The differentially binding peaks were identified by a python script, using fisher test.

Data quality

To ensure that our data were of high quality and reproducibility, raw sequencing data was first filtered by Trimmomatic (version 0.36), low-quality reads were discarded and the reads contaminated with adaptor sequences were trimmed.

Software

Raw sequencing data was first filtered by Trimmomatic (version 0.36). The clean reads were mapped to the mouse genome using STAR software (version 2.5.3a). The RSeQC (version 2.6) was used for reads distribution analysis. The MACS2 software (Version 2.1.1) was used for peak calling. The bedtools (Version 2.25.0) was used for peaks annotation and peak distribution analysis.
